# Supplementary material for: Are carbon emissions trading and green financial instruments synergistic? -Comprehensive quantitative research based on content analysis
Source: PLoS One. 2024 Mar 7;19(3):e0298601. doi: 10.1371/journal.pone.0298601 (PMC10919601; doi:10.1371/journal.pone.0298601)
Supplement: S1 Data — (ZIP) [file pone.0298601.s001.zip › Data/地级市Z_F工作报告.zip!/╣π╢1⁄2/╒╪╟∞╩╨/└┤╫╘╣1⁄2╓┌║┼í╛┬φ┐╦╩2╛▌═°í┐_2015.docx]

各位代表：

　　我代表肇庆市人民政府向大会作工作报告，请予审议，并请政协委员和其他列席人员提出意见。

　　2014年工作回顾

　　去年，面对复杂严峻的经济形势，在省委、省政府和市委的正确领导下，在市人大及其常委会和市政协的监督支持下，市政府全面贯彻党的十八届三中、四中全会和习近平总书记系列重要讲话精神，紧紧围绕建设成为珠三角连接大西南枢纽门户城市的奋斗目标，全面落实《珠三角规划纲要》，深入实施“两区引领两化”战略，着力稳增长、促改革、调结构、惠民生，基本实现经济社会发展预期目标，全部工业总产值超4千亿元，第二产业占三大产业比重首次突破50%，粤桂合作特别试验区上升为国家战略，肇庆新区获得省政府首批9.3亿元股权投资基金，南广、贵广铁路建成通车，肇庆进入“高铁时代”。

　　一、突出抓发展调结构，经济实现稳中有进

　　经济保持平稳增长。大部分主要经济指标增速居珠三角前三位和全省中上水平。全年实现地区生产总值1845.06亿元，比上年增长（以下简称增长）10%，人均生产总值45795元，增长9.3%，一、二、三产业增加值分别增长4.3%、14%、7%；完成地方公共财政预算收入139.1亿元，增长15.2%；完成固定资产投资1138.73亿元、社会消费品零售总额559.9亿元、外贸进出口总额78.4亿美元，分别增长19%、13.5%、11.8%。居民消费价格指数上涨2.7%，城镇登记失业率为2.36%。县域经济持续发展壮大，四会市和高要市在全省县域经济综合发展力排名中分别名列第一、第五位，四个山区县的地区生产总值和规模以上工业增加值增速均达到或超过全市平均水平。

　　三大产业加快发展。三次产业比调整为14.7:50.0:35.3。工业规模效益上新水平，完成规模以上工业增加值932.42亿元，增长14.3%，增速居珠三角首位；规上工业企业实现税收增长超20%。我市被纳入珠江西岸先进装备制造产业带，九大主导产业增加值占比提高到65.6%，金属加工产业成为首个年产值超千亿元的产业集群。年主营业务收入超10亿元企业达68家，累计有21家企业被认定为省高成长性中小企业，怀集登云汽配成为我市山区县首家上市公司，羚光电子成为全市首家“新三板”上市企业。现代服务业增加值占服务业比重达45%，服务外包离岸执行金额增长55%；金融机构本外币各项贷款余额比年初增长11.5%，融资超市为中小微企业提供贷款163.29亿元；旅游综合改革示范市启动建设，旅游收入增长7.4%；限额以上单位通过互联网实现销售增长76.7%，汽车销售增长 36.1%；房地产投资增长10.1%；港口货物吞吐量突破3000万吨。农林牧渔业增加值完成272.03亿元。高标准基本农田年度建设项目全面完成，获省奖励用地指标1550亩。粮食生产实现六连增，新品种水果种植、畜牧产品和水产品养殖迈向高值化。广东省（高要）粤台农业合作试验区获批设立。农业龙头企业增至178家，农民专业合作社总量居全省第三，分别带动14.8万、13.4万农户户均增收近3000元。

　　结构调整扎实推进。建立重点技术创新和改造项目库，实施工业技改项目107个,总投资近50亿元。先进制造业增加值和高新技术产品产值占比分别达到31.1%、27.1%。新认定高新技术企业9家、省创新型企业3家、省工程技术研究中心16家，新增省级高新技术产品121项，发明专利申请量和授权量分别增长35.9%、25.9%，全面完成省部产学研结合示范市建设任务。建成国家级建筑五金产品质检中心和市电子信息、铝合金压铸产品质检平台，新增名牌产品30个、驰名商标7个。工业化和信息化加快融合，建成创意产业工程中心等公共创新平台和华南智慧城等物联网、云产业基地。单位生产总值能耗下降2%，提前完成“十二五”淘汰落后产能任务，广宁县成为省循环经济示范县。

　　重点项目建设提速。超额完成重点项目年度投资计划，100项市重点项目完成投资439.32亿元,其中省重点项目完成年度投资136.1%，完成率全省第一；交通重点项目完成投资165.98亿元,创历史新高。又一条出省通道二广高速怀集至连州段建成通车，直通广州白云机场的珠三角环线高速黄岗至花山段建成使用，广佛肇城际轨道肇庆段完成路基建设，广佛肇高速公路全线施工，汕昆、汕湛高速肇庆段正式动工，枢纽门户城市的立体交通网架逐步成型。现代产业项目完成年度投资 145.3%，中恒一期、风华高端新型电子、三浦重工等重点产业项目投产。景福围三四期等重点水利工程进展顺利。

　　二、突出抓平台促开放，发展后劲持续增强

　　产业园区扩能增效。肇庆高新区生产总值增速居全市首位，新增注册企业增长63.4%，新投产企业25家，新动工和增资扩产项目42个，引进海王集团等优质项目57个，被认定为省市共建智能制造示范基地，被评为“中国最佳营商环境十大园区”，大旺产业园连续六年被评为省优秀园区。德庆产业园完成规划调整，高要产业园申报获批，肇庆（高要）汽车零部件产业园16个在建报建项目总投资近40亿元。全市承接产业转移项目85个，合同总投资近200亿元，三个省级产业园规上工业增加值和税收分别增长22.9%、20.6%。

　　区域合作不断深化。广佛肇经济圈建设深入推进，实施合作项目22个，广佛肇（怀集）经济合作区上升为省级发展战略，起步区路网管网建设基本完成，累计引进项目64个,已建成投产14个。肇梧战略合作取得新突破，38项重点合作项目启动实施，粤桂合作特别试验区管委会组建运作，连接两广交界的大园桥等项目建成，引进项目17个。与云浮、贺州等周边地区合作迈出新步伐。

　　招商引资质量提升。出台创新招商引资行动计划，大力实施主题招商、产业招商，成功举办“珠三角—东盟·大西南经贸合作交流会”和“肇庆金秋—先进装备制造业招商洽谈会”，分别引进投资685亿元、496亿元。全年新批外商投资项目107 个，合同外资额33.3亿美元，增长7%，新批和增资投资额千万美元以上项目97个；实际吸收外资13.3亿美元，增长7.4%。

　　三、突出抓建设强治理，城乡发展统筹推进

　　新型城市化稳步推进。出台“多规融合”的《“两区引领两化”发展总体规划》，新一轮城市总体规划启动编制，中心城区规划范围从194平方公里扩大到500平方公里。肇庆新区城市总体规划获省批准实施，起步区建设全面启动，累计收储土地2.7万亩，已建或在建道路11条，北师大附属学校、肇庆东站等重点项目建成，引进环球金融中心、中央绿轴生态城等优质项目。中心城区一体化发展加快，阅江大桥、西江北岸江滨西路示范段、鼎湖大道等项目进展顺利，新动工及续建“三旧”改造项目15个。各县(市)开展了新一轮总体规划修编，新城建设全面启动。智慧城市建设加快，城市互联网普及率达62.4%，光纤覆盖95%以上城市小区、通全部行政村。

　　新农村建设步伐加快。市域乡镇总体规划实现全覆盖，编制完成100个行政村规划，村庄规划率提升到80%。名村、示范村创建进度和质量走在全省前列，绿化美化乡村141条，新创建省卫生镇6个、省卫生村173条、生态文明示范村29条，累计创建省级宜居城镇21个、宜居村庄39条。镇村全部建成垃圾转运站和收集点。

　　生态建设成效显著。掀起新一轮绿化肇庆大行动和国家森林城市创建热潮，国家园林城市通过省复查。完成植树造林29.64万亩，建成生态景观林带160.8公里，新增森林公园24个、湿地公园2个，北岭山森林公园建成开放。市垃圾场扩容二期工程和县级垃圾无害化处理场建成使用。划定乡镇以上饮用水源保护区，完成重点流域水环境综合整治和星湖水质整治首期工程，生活污水处理率提高到86.1%。

　　大气整治力度加大。专门成立环境保护委员会和组建环保警察，实施环保六大行动计划，实行环境监察网格化管理，关停违法违规排放企业117家，主要污染物总量减排年度任务全面完成。全力推进大气污染防治，重点加强工业燃煤、机动车尾气、扬尘等污染综合治理和环保综合执法，63条陶瓷企业生产线改用天然气、206条签订用气协议，陶瓷企业全面安装在线监控系统，整治锅炉244台，淘汰黄标车及老旧车3.58万辆。全年空气质量优良率上升2.5个百分点，PM2.5、PM10浓度年均值逐步下降，完成省政府下达的改善环境空气质量年度考核目标任务。

　　四、突出抓实事惠民生，社会事业全面发展

　　民生保障不断完善。公共财政用于10类民生支出163.69亿元，占预算支出69. 5%。十件惠民实事顺利完成，提升就业水平、保障性住房建设、农村公路硬底化等超额完成任务。各类社保参保任务全面完成，基本医疗保险覆盖常住人口，社会保障卡发放覆盖参保人群。城乡居民基础养老金提高到每月80元，企业退休人员基本养老金提高到月均1801元，鼎湖区被征地农民养老保障经验在全省推广。城乡居民大病保险年最高支付额提高到27万元。家庭年人均收入低于3600元的农村居民、低于5640元的城镇居民和城镇“三无人员”全部纳入低保。五保供养标准达上年农村居民人均纯收入60%，发放高龄老人津贴4280万元。累计投入扶贫“双到”资金4.55亿元，贫困户年人均纯收入提高到6198元。城乡居民收入与经济增长基本同步。

　　社会事业蓬勃发展。省教育强镇创建任务全面完成，端州区通过教育现代化先进区督导验收，6个县（市、区）通过全国义务教育发展基本均衡督导评估，公办义务教育学校全部建成标准化学校，在公办学校接受义务教育的进城务工人员随迁子女占比高出全省平均水平27.3个百分点,广东理工学院升格为本科院校。6个县（市）全部纳入县级公立医院综合改革试点，基层医疗卫生机构100%配置使用基本药物，完成首批乡镇卫生院“五个一”设备装备，98.9%的乡镇卫生院提供中医药服务，有效防控登革热等疾病。包公文化园主体工程基本完成，“三馆一宫”启动规划建设，成功承办省第六届群众音乐舞蹈花会，四个山区县被评为(复评)全国文化先进县，建成100个村级文化站电子阅览室，开展文化管理体制试点改革。启动奥体中心和50个社区体育公园建设，提前实现乡镇农民体育健身工程全覆盖。受理“单独二孩”申请5167例。统计、民族、宗教、外事、侨务、港澳、人防、国防动员、地方志、档案、气象、水文、地震、慈善、妇女儿童、残疾人等工作取得新成绩。

　　社会管理不断完善。实施了城市管理综合行政执法改革，城管“执法通”防控平台推广应用。城市社区综合服务设施覆盖率达90%，端州区被评为全国和谐社区建设示范城区。建立诚信“红黑榜”发布制度。出台第二批市本级社会组织承接政府职能转移和购买服务目录，新增社工机构5家、社会组织144家。社会治安“大视频”建设有效推进，联勤武装巡逻实现常态化，“八类”严重暴力犯罪案件下降17.1%，各类矛盾纠纷调解成功率达97.7%。安全生产、食品药品安全形势总体平稳。

　　五、突出抓改革提效能，政府自身建设不断加强

　　体制改革深入实施。市政府牵头组织的45项年度改革任务40项已经完成、5项正在报批。国资国企改革加快推进，组建国联控股新型融资平台公司，水务集团引入了战略投资者。商事制度、企业投资备案制等改革基本完成，新企业设立审批实现“一表登记、并行审批、三证同发”，新登记企业及注册资本（金）分别增长46.4%和163.8%。高新区和新区出台投资鼓励清单，新区探索实施“资金融通+产业导入”城市建设投融资模式并搭建起“两级土地股份化”运营平台。德庆县成为中央农办农村改革试验联系点，探索实施农村金融抵押贷款新模式，率先完成农村土地承包经营权确权登记颁证试点工作。引进了市场主体参与国有林场生态旅游和生态公益林建设。

　　行政效能不断提升。市县政府机构改革全面完成，职能转变稳步推进，3个试点县区和10个部门向社会公布了权责清单，高要市金利镇成为省行政管理体制改革试点。行政审批实施标准化服务，审批事项精简率达43%，进驻网上办事大厅的审批事项达一、二、三级深度的分别占100%、90%、52%，社会服务事项基本实现在线申办。高新区和新区试点实施行政审批超时默许制。市县两级设置公共资源交易机构，设立农村“三资”管理服务平台和交易服务中心的乡镇分别占总数的100%、83%。

　　法治廉政建设深入推进。认真落实向市人大及其常委会报告工作制度，按期办理人大代表建议和政协委员提案。扎实推进政府系统党的群众路线教育实践活动，修改完善了《政府工作规则》等制度，161项整改任务全面完成。开展整治“四风”问题十个专项行动，组织明查暗访323次。认真落实中央“八项规定”精神，“三公”经费支出降低25.4%，清退超标超配公车86辆，调整多占办公用房14.1万平方米，政府系统会议、文件分别减少31.3%和37.5%，市级以下评比达标表彰活动全部停止。严格执行市政府重大行政决策程序规定，行政机关负责人出庭应诉机制和重大行政处罚备案制度有效落实。完成一村（社区）一法律顾问配备，县镇村三级公共法律服务实体平台启动建设。廉政监督、审计监督和效能监察不断加强，“市民问政”工作实现常态化和规范化。严格执行信访条例，市领导共接访1727批次4399人次。

　　过去一年政府工作取得的成绩，是全市人民和社会各界凝心聚力团结奋斗的结果。我代表市人民政府，向全市人民，向驻肇部队和各级驻肇单位，向所有关心支持参与肇庆建设发展的各界人士致以崇高的敬意和衷心的感谢！同时，我们也清醒认识到，我市发展仍然存在不少困难和问题：受经济大环境总体下行和统计政策调整影响，生产总值、规模以上工业增加值增幅未能实现预期目标，人均生产总值与全国平均水平仍有差距；具有核心竞争力和较强带动能力的企业不多，先进装备制造业仍然薄弱；服务业面临下行压力，外贸出口形势仍然严峻；产城融合互动发展力度不足，新型城市化步伐有待加快；空气质量改善还不够明显，环境综合治理的长期性和艰巨性与人民群众对良好环境迫切需求之间的矛盾越发突出；基本公共服务水平有待提高，政府自身建设仍需加强。对此我们将高度重视，采取更有针对性的措施加以解决。

　　2015年工作安排

　　今年是冲刺“十二五”、谋划“十三五”的重要一年。习近平总书记作出了关于经济发展新常态的重要论述，国家全面加快建设21世纪海上丝绸之路、着力打造中国—东盟自贸区升级版，我省大力实施《珠江—西江经济带发展规划》、积极推动广东自由贸易试验区建设、加快发展珠江西岸先进装备制造产业带，为我市建设枢纽门户城市提供强大动力。做好今年的政府工作，必须准确把握新常态下的新特征，主动抢抓新机遇，努力开创新局面。今年政府工作的总体要求是：贯彻落实党的十八届三中、四中全会精神及省委、省政府和市委的决策部署，立足经济发展新常态，深入落实《珠三角规划纲要》和《珠江—西江经济带发展规划》，大力实施“两区引领两化”战略，坚持发展第一要务，切实把深化改革开放和调整经济结构贯穿于发展各个领域，更加注重提升发展质量，更加注重产城融合互动，更加注重保护生态环境，更加注重保障改善民生，更加注重法治政府建设，全面完成“十二五”规划目标任务，为加快建设珠三角连接大西南枢纽门户城市打下坚实基础。今年经济社会发展主要预期目标是：地区生产总值增长10.5%左右，人均生产总值增长9.5%，一般公共预算收入增长12%，固定资产投资增长16%，规模以上工业增加值增长14%，社会消费品零售总额增长12%，实际吸收外资增长4%，外贸进出口总额增长3%，居民消费价格涨幅控制在3%左右，居民收入增长与经济增长基本同步，城镇登记失业率控制在2.6%以内，人口出生率和自然增长率、节能减排和空气质量改善目标控制在省的计划以内。重点抓好以下工作：

　　一、推动经济持续发展，全面实现“十二五”目标。充分发挥投资、消费和出口的拉动作用，牢牢把握交通基础设施建设、产业平台创新发展、城市扩容提质三大重点，积极培育新的经济增长点。着力扩大项目投资，全年安排重点项目100项，计划投资373.12亿元。着力扩大消费需求，组织实施六大领域消费工程，推动“电商进村”、“快递下乡”和“新网工程”，发展线上线下融合的电商服务模式；加快推进星湖景区创5A工作，有序开发西江旅游资源，提升旅游接待服务水平；促进房地产、商贸等行业稳定健康发展。着力推动外贸转型发展，支持跨境电商拓展“肇货出口”，发展壮大服务贸易和服务外包产业。全力抓好实体经济，组织实施新一轮技术改造，力促企业增资扩产、达标达产，支持企业利用多层次资本市场融资发展，重点扶持50家可在三年内实现产值或税收翻番企业，促进高成长性中小企业倍增发展。积极打造高铁经济带，加快谋划实施城轨沿线站点综合开发。做大做强航运经济，协同推进西江、北江扩能升级工程，加快发展港口物流业。科学谋划“十三五”，做好规划编制，力促更多重点项目进入国家和省的规划笼子。

　　二、深化重点领域改革，增创体制机制新优势。全面落实市委确定的年度重点改革任务，抓紧推进一批改革试点。深化行政审批制度改革，完成非行政许可审批事项清理工作，全面落实政府部门权责清单制度，推进行政审批标准化建设。深化商事制度改革，完善市级公共信用信息管理系统和公示系统。进一步健全政府预算体系，加快清理整合各类专项资金，全面公开财政预决算和部门预决算信息。扩大公共资源交易管理范围。深化投融资体制改革，扩大政府债券融资规模，以探索实施股权投资、PPP投资模式为重点，创新重大发展平台开发建设投融资方式，扩大民间资本参与基础设施、公益项目的投资领域，加快引进创投、风投等股权投资。抓好四会、德庆普惠金融创新试点。积极发展民生金融。深入推进市属国资国企整合，大力实施产权多元化改革。继续推进综合执法体制改革，加快理顺市、区关系，更好地落实属地化管理。深化农村综合改革，全面铺开农村土地承包经营权确权登记颁证工作，促进集体建设经营性用地使用权规范有序流转。加快推进供销社综合改革试点。继续深化教育、医药卫生、文化等民生公共服务供给机制改革。

　　三、着力培育主导产业，加快结构优化升级。坚持走新型工业化发展道路，积极协同打造珠江西岸先进装备制造产业带，重点发展汽车及零部件制造、电子信息、智能制造、节能环保等优势产业，加快广汽集团汽车零配件等项目建设；积极发展战略性新兴产业，支持风华高科新材料等项目扩大发展，加快推进海王、康芝等生物医药项目；集聚发展资源深加工产业，发展壮大高要金鼎、怀集铁矿、德庆稀土等矿业项目。提升现代服务业发展水平，大力实施“光网城市”工程，促进新一代信息基础设施共建共享，积极打造电子商务产业园区和产业集聚培育区，加快发展软件信息、互联网金融等新业态。抓好创新型城市和国家知识产权试点城市建设，加强产学研协同创新，促进金融科技产业融合发展，推进长春理工大学国家大学科技园肇庆分园、肇庆学院大学科技园等科技平台建设，支持企业创建重点实验室。深入实施品牌带动和质量强市战略。

　　四、推进城市扩容提质，促进产城融合发展。坚持以肇庆新区引领新型城市化，加快打造珠三角城市群重要门户。全面推进县（市）“多规合一”，编制新型城镇化规划实施意见和县（市）全域城乡建设规划。加快搭建肇庆新区城市主体框架，高标准抓好起步区基础设施建设，推进市文化馆新馆、市档案馆新馆、新规划馆和市少年宫等项目，抓好环球金融中心、中央绿轴生态城、国际商贸城、临港物流园四大产业平台建设和招商。稳步推进中心城区区划调整，强化“一江两岸”统一规划建设，抓好阅江大桥、国道城市化改造等城际交通项目和江滨堤路、建设二路等城市路网改造工程，启动七星岩北门广场规划建设，加快推进“三旧”改造。大力发展城市公共交通，完善城区天然气站和管网建设，加强城市地下管线建设管理。加快县域城市新区拓展建设，组织实施四会大沙镇新型城镇化改革试点和高要金利镇中小城市综合改革试点。健全流动人口居住证制度，推进城镇基本公共服务常住人口全覆盖。

　　五、加快平台项目建设，强化引领带动能力。扎实推进肇庆高新区现代科技工业城和智能制造示范基地建设，着力引进“专精特新”项目，加快落实省微生物研究所工业生物六大孵化平台等科技创新项目。力争粤桂合作特别试验区实施东西部特殊政策，抓好规划衔接、用地储备和联合招商，推动广梧高速封开连接线拓宽等基础设施项目规划建设。继续推进广佛肇（怀集）经济合作区起步区建设，促进企业入园发展。加快产业转移园区扩能增效，做大做强汽车、精细化工、再生资源等一批专业园区。积极开展主题招商、产业招商，培育发展总部经济，力争引进世界500强、中国500强和行业前10位企业的重大项目。全力抓好交通能源重点项目建设，确保广佛肇城际轨道肇庆段建成通车，加快广佛肇高速、汕昆高速和汕湛高速肇庆段以及火车站综合体、新客运总站等交通枢纽建设，力争怀阳高速肇庆段列入省重点前期预备项目，协调推进中电四会热电、德庆大顶山风电和封开党山抽水蓄能电站等重点能源项目建设。

　　六、大力发展现代农业，提升“三农”发展水平。加快实施现代农业发展五年计划，切实转变农业发展方式。加大农业基础设施投入，完成24.8万亩高标准基本农田建设，抓好景福围三四期、病险水库加固、小河流域治理、村村通自来水等重点水利工程，推进“菜篮子”基地、养殖场、现代标准化鱼塘等设施建设，提高农业机械化水平，推行农业清洁生产。稳步提升传统农业，促进粮食增产增收。壮大特色效益农业，扶持发展蔬菜水果、花卉苗木、水产养殖等特色优势产业和农产品加工业，加快农产品产地市场建设，培育农业名牌产品。积极构建新型农业经营体系，实施新型职业农民培育工程，大力发展家庭农场、专业大户、农民专业合作社和农业龙头企业，高标准建设广东省（高要）粤台农业合作试验区。继续完善县、镇农检站网络建设，加强农产品认证、质量安全监管和重大动植物疫病防控，推广政策性涉农保险。推动农村政务服务、土地流转等平台全覆盖。加快建设宜居城镇村庄、卫生镇村和生态文明村，推进省级新农村示范片建设。完成新一轮扶贫“双到”任务。

　　七、狠抓环境综合治理，努力建设美丽肇庆。全面贯彻落实新《环境保护法》，抓好环保严防、严管、严惩，打好环境空气质量综合治理攻坚战。严格落实主体功能区规划，编制实施《生态文明建设规划》，划定生态控制线和林业生态红线，建立差别化环保准入制度。严格实施污染物排放总量控制，深入推进环境污染第三方治理，落实环境监察网格化管理和环保警察联动执法机制，严厉打击环境违法行为。重点抓好大气污染防治，强化陶瓷行业和工业锅炉综合治理，加快黄标车和老旧车淘汰，落实扬尘源防控措施。坚持最严格水资源管理，启动建设高新区第二污水处理厂和市第三污水处理厂二期扩建，加快星湖水质整治二期工程。全面完成县（市）垃圾填埋场建设，建立完善农村生活垃圾收运体系，开展生活垃圾分类试点。推进节能减排重点工程，加强工业、交通、建筑、公共机构等重点领域节能，推广应用新能源汽车，坚决淘汰落后产能。全力创建国家森林城市，加快生态景观林带、森林公园、湿地公园建设和乡村绿化美化，启动羚山古栈道文化森林公园规划建设。做好国家卫生城市复查迎检。加强土地集约节约高效利用，建设全市“一张图”核心数据库，严厉查处违法违规用地。加强地质灾害防治，推进绿色矿山建设。

　　八、扎实做好民生保障，全面发展社会事业。健全公共就业创业服务体系，力争端州区社区公共就业服务平台覆盖率达85%以上，重点促进高校毕业生、农村转移劳动力、城镇困难人员和退役军人就业。大力推动大众创业，组织实施大学生创业引领计划。完善劳动监察“两网化”和单位用工信息数据库。加大社会保险扩面力度，推进企业全员足额参保，稳步推进机关事业单位养老保险制度改革，全面实现城乡居民医保市内就医即时结算，加快实施城乡居民医疗保险引入市场机制试点和大病保险。扩大社区和居家养老服务覆盖面。完善住房保障体系，健全保障性住房用地供应规划、建设和分配机制。推动全国义务教育发展基本均衡县（市、区）实现全覆盖，创建教育现代化先进区（市），实施第二期学前教育三年行动计划和特殊教育提升计划，支持肇庆学院“申硕改大”、肇庆医专和广东工商职业学院“专升本”，创新职教办学模式，加快发展民办教育。深化医药卫生体制改革，全面推进公立医院综合改革，鼓励社会办医，加快中山大学附属肇庆医院等重点项目建设，全面完成乡镇卫生院“五个一”设备装备，加强重大疫情监测和防治，推进中医药强市建设。加强计划生育服务管理，提升妇幼健康服务能力。加强社会主义核心价值观教育，继续抓好全国文明城市创建工作。推进公共文化服务标准化建设，大力实施文化惠民工程，办好群众文化活动，创新文化社会团体管理体制，抓好文化产业招商，鼓励发展非公有制文化企业，打造一批文化产品交易平台，加快市博物馆新馆、包公文化园、黎雄才美术馆等重点文化设施建设。广泛开展全民健身活动，规划建设社区体育公园30个，加快奥体中心建设。完善社会救助和防灾救灾体系，创建“全国综合减灾示范社区”。健全食品药品安全监管网络。强化安全生产监管和标准化建设。加强社会管理和服务，积极预防和有效化解社会矛盾，严厉打击违法犯罪，全面推进平安肇庆建设。努力争创双拥模范城。加强统计、民族、宗教、外事、侨务、港澳、人防、国防动员、地方志、档案、气象、水文、地震、慈善、妇女儿童、残疾人等工作。

　　各位代表，今年是全面推进依法治国方略的开局之年，市政府将按照中央和省的部署，贯彻落实市委关于全面推进依法治市的实施意见，加快建设法治服务型政府。一是强化依法民主决策。自觉接受人大及其常委会法律监督和政协民主监督，定期向人大及其常委会报告工作和向政协通报情况，高质量办好人大代表建议和政协委员提案。认真听取各民主党派、工商联、无党派人士、人民团体的意见建议。做好地方立法的服务与配合工作。健全依法决策机制，完善重大行政决策合法性审查制度，试行重大行政决策风险评估、实施后评估、终身责任追究和责任倒查制度。二是提高依法行政水平。依法有序全面履行政府职能，推动行政机关机构、职能、权限、程序、责任法定化。全面推进公共资源配置、重大建设项目实施、社会公益事业建设等重点领域的政务信息公开，完善政府和部门门户网站、微博、微信等公开规范建设。完成“六五”普法任务，完善各县（市、区）政府法律顾问制度，构建公共法律服务体系。三是创新行政服务方式。拓展网上办事大厅三级办理深度，推广网上申请和“一表制”改革，提升网上办理率和办结率，积极推进网上办事服务向基层延伸。继续向基层下放事权，培育发展社会组织，大力推行政府购买社会服务。四是抓好廉政勤政建设。深入落实中央“八项规定”等系列文件要求，切实巩固党的群众路线教育实践活动成果，持之以恒纠正“四风”，严控“三公”经费支出，完成公务用车制度改革。完善惩治和预防腐败体系，强化行政监察、审计监督和专项审计整改，加强对重点领域、重点项目、重点工作、关键环节的廉政监督。进一步畅通信访渠道，健全领导定期接访下访包案制度。

　　各位代表，在落实省十件民生实事有关任务的同时，市政府将全力办好今年十件惠民实事：一是提高底线民生保障。二是促进教育均衡发展。三是改善医疗卫生服务。四是落实保障住房建设。五是扩大就业创业培训。六是加强困难群体帮扶。七是加强生态环境治理。八是加快城乡道路建设。九是优化公共文体服务。十是完善公共法律服务（具体内容见附件）。

　　各位代表，经济发展新常态为我市加快科学发展带来新的战略机遇。让我们紧密团结在以习近平同志为总书记的党中央周围，按照省委、省政府和市委的决策部署，抢抓机遇，锐意改革，真抓实干，开拓进取，为加快建设成为珠三角连接大西南枢纽门户城市而努力奋斗!
